# Isolated splenic littoral cell angioma in a child: a case report

**DOI:** 10.3389/fonc.2026.1770088

**Published:** 2026-06-16

**Authors:** Huihui Lin, Xiaoyu Wang, Longxiu Zhang

**Affiliations:** Department of Radiology, Anhui Provincial Children’s Hospital, Hefei, China

**Keywords:** computed tomography (CT), pathological diagnosis, pediatric, splenic littoral cell angioma (LCA), surgical resection

## Abstract

We report a case of isolated splenic littoral cell angioma (LCA) in a 9-year-old male child, who was admitted for surgical treatment after an incidental discovery of a splenic space-occupying lesion during a physical examination one year prior at an external hospital(no significant reduction on follow-up). The child had no specific clinical symptoms, with only a slightly elevated platelet count (454×10^9^/L) and normal results of other laboratory and auxiliary examinations. Abdominal computed tomography (CT) showed a round-like hypodense mass (4.8 cm×4.8 cm×5.3 cm) in the upper pole of the spleen (plain CT value: ~43 HU). On enhanced CT, the mass exhibited characteristic “fireball-like” enhancement in the arterial phase (CT value: ~182 HU), continuous progressive enhancement in the portal venous phase (CT value: ~133 HU), and uniform density with mild attenuation in the delayed phase (CT value: ~98 HU), remaining slightly hyperdense relative to the normal spleen. Laparoscopic partial splenectomy was performed, and pathological examination combined with immunohistochemistry (CD31+, CD34+, CD68+, CD8+, SMA+, CD163+; Ki67: ~7%; D2-40-, CD21-, CD3-, CD20-) confirmed the diagnosis of LCA. No recurrence was observed at 3-month and 6-month postoperative follow-up. LCA is a rare primary splenic vascular tumor, especially uncommon as an isolated lesion in children. Its clinical manifestations are nonspecific, and CT enhancement features have important indicative value. Definitive diagnosis relies on pathology, and surgical resection yields a favorable prognosis.

## 1 Introduction

Littoral cell angioma (LCA) is a rare primary vascular tumor of the spleen, first described by Falk et al. in 1991, originating from the littoral cells of the splenic red pulp (1). Splenic littoral cell tumors are categorized into three subtypes with identical immunophenotypes: LCA, littoral cell hemangioendotheliomaand littoral cell angiosarcoma. The biological behavior of LCA remains controversial—some studies suggest malignant potential, while recent evidence reclassifies it as a benign primary splenic vascular tumor, possibly representing an intrasplenic manifestation of systemic dysfunction (2).The cause of LCA is still unclear. However, LCA has been observed to occur more frequently in pediatric patients with immunodeficiency (3). Clinically, LCA predominantly affects adults, with multiple lesions being more common than solitary ones. Isolated LCA in pediatric patients is extremely rare, with most sporadic case reports globally. Most patients are asymptomatic and diagnosed incidentally, leading to frequent misdiagnosis or missed diagnosis.

Given the paucity of pediatric cases, the clinical, imaging, and prognostic characteristics of isolated pediatric LCA remain not fully elucidated. Herein, we report a case of isolated splenic LCA in a 9-year-old boy, supplemented by literature review, to enhance understanding of this rare disease and provide reference for clinical practice.

## 2 Case presentation

A 9-year-old male child was admitted to Anhui Provincial Children’s Hospital for surgical treatment. One year prior, a space-occupying lesion in the upper pole of the spleen was incidentally detected during a routine physical examination at an external hospital, and follow-up imaging showed no significant reduction in lesion size. The child had no specific symptoms such as abdominal pain, vomiting, distension, fever, or chills. Physical examination revealed no jaundice of the skin or sclera, and abdominal palpation showed no tenderness or mass.

Laboratory investigations: Platelet count was 454×10^9^/L (reference range: 100–300×10^9^/L); urinalysis, liver and kidney function, coagulation function, chest X-ray, and electrocardiogram were all within normal ranges. Abdominal ultrasound indicated a solid hypoechoic mass in the upper pole of the spleen with abundant blood flow signals, suggesting a hemangioma.

**Figure 1:**
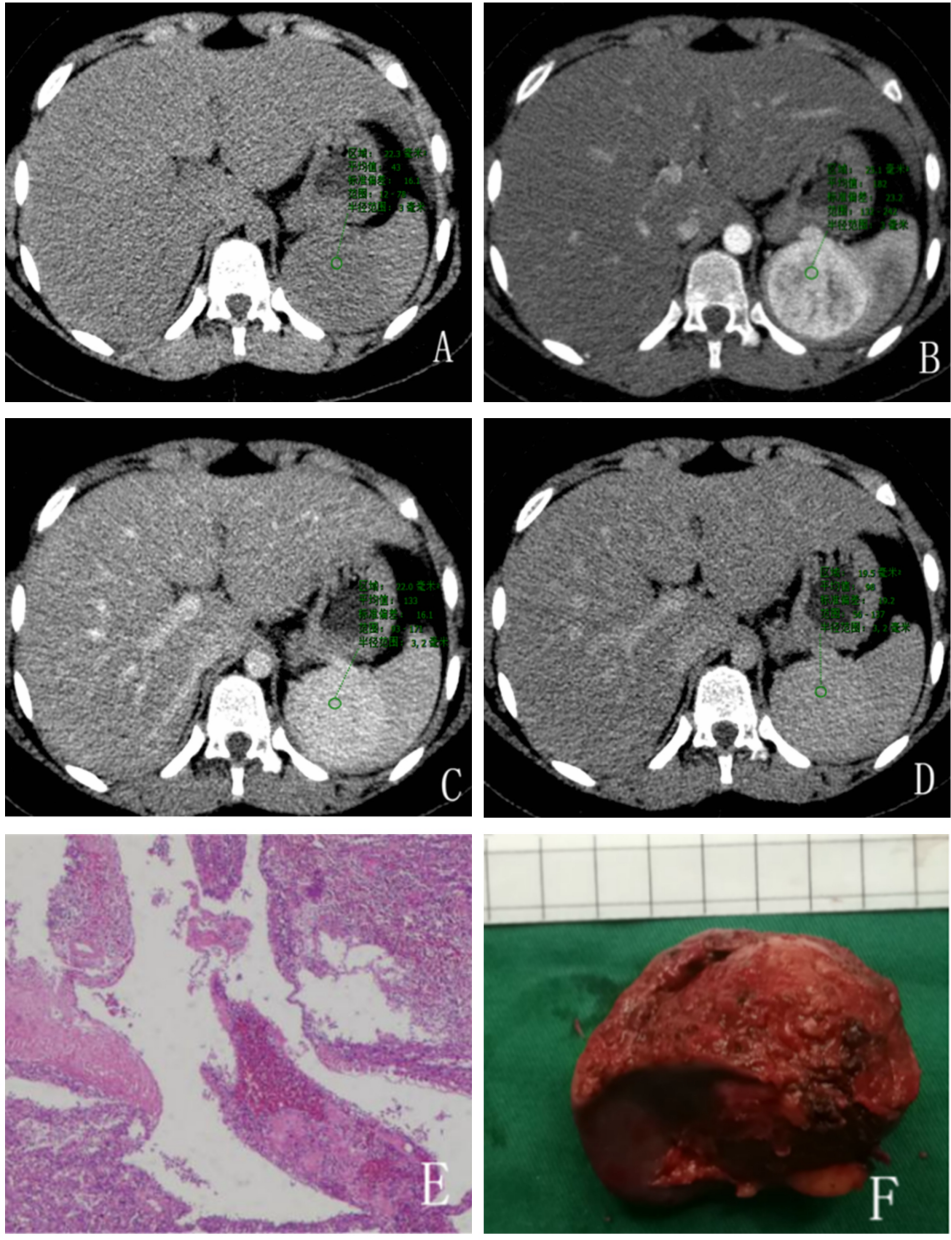
Imaging, pathological, and gross findings of the 9-year-old male with isolated splenic LCA. **(A)** Axial CT plain scan: A round-like hypodense lesion in the splenic upper pole (average CT value: ~43 HU) with a clear boundary, partially protruding anteromedially. **(B)** Arterial phase contrast-enhanced CT: Obvious “fireball-like” enhancement (CT value: ~182 HU) with multiple patchy hypodense areas, clear demarcation from adjacent tissues. **(C)** Portal venous phase contrast-enhanced CT: Centripetal progressive enhancement (CT value: ~133 HU) with relatively uniform density, slightly higher than normal spleen. **(D)** Delayed phase contrast-enhanced CT: Uniform enhancement with mild attenuation (CT value:~ 98 HU), still hyperdense to normal spleen. **(E)** Pathological section (HE staining, ×100): Anastomosing dilated vascular lumens with papillary projections. **(F)** Gross specimen: Grayish-red solid mass with a clear boundary.

Abdominal CT plain scan and enhanced imaging (**Figures 1A–D**) demonstrated that the spleen was larger than 5 costal units. A round-like hypodense mass (4.8 cm × 4.8 cm × 5.3 cm) was identified in the upper pole of the spleen, with a clear boundary and a plain CT value of approximately 43 HU, partially protruding anteromedially. On arterial phase contrast-enhanced CT, the mass showed obvious heterogeneous enhancement (CT value: ~182 HU), which was slightly lower than the abdominal aortic blood pool (205 HU) and significantly higher than the adjacent abdominal wall skeletal muscle (65 HU), resembling a “fireball” with multiple small patchy hypoenhanced foci inside and clear demarcation from adjacent tissues.”Fireball-like enhancement” refers to the obvious heterogeneous high enhancement of the tumor in the arterial phase of contrast-enhanced CT (CT value increased by more than 130 HU compared with plain scan), with multiple small patchy hypoenhanced foci in the center, showing a fireball-like morphological feature on axial images. During the portal venous phase, continuous centripetal progressive enhancement was noted, with a CT value of 133 HU and relatively uniform density (a few patchy hypoenhanced areas remained), slightly higher than normal splenic parenchyma. In the delayed phase, the enhancement degree slightly decreased (CT value: ~98 HU), but the density remained slightly higher than the normal spleen, with uniform enhancement. Thus, in the portal venous phase and delayed phase, the tumor attenuation was close to the splenic vein blood pool (135 HU/95 HU) and persistently higher than skeletal muscle. CT preoperatively diagnosed a hypervascular tumor originating from the spleen, with hemangioma as the primary consideration.

Laparoscopic partial splenectomy was performed under general anesthesia. Intraoperatively, a 4.5 cm × 4.5 cm × 5.5 cm mass was found in the upper pole of the spleen, with a clear boundary, grayish-red color, and blood supply from the splenic artery. Partial splenectomy was conducted with a 1.5 cm margin from the tumor boundary to ensure complete resection.

Pathological examination (**Figures 1E, F**): Gross specimen showed a grayish-red solid mass with a clear boundary and intact capsule. Microscopically (HE staining, ×100), the tumor was composed of anastomosing vascular lumens with varying degrees of dilation, accompanied by papillary projections. Immunohistochemical results: CD31 (+), CD34 (+), CD68 (+), CD8 (+), SMA (+), CD163 (+); Ki67 proliferation index was approximately 7%; D2-40 (-), CD21 (-), CD3 (-), CD20 (-). Based on morphological and immunophenotypic features, the diagnosis of splenic littoral cell angioma (LCA) was confirmed.

Postoperative recovery was uneventful, and the child was discharged 5 days after surgery. Ultrasound follow-up at 6 months and 1 year postoperatively showed no recurrence or complications such as sepsis or thrombosis.

## 3 Discussion

### 3.1 Clinical and etiological characteristics

LCA is a rare primary splenic vascular tumor. It predominantly occurs in middle-aged and elderly adults (mean age: 50~60 years), with no significant gender predilection (2). Isolated LCA in children is extremely rare, and to date, only a few cases have been reported in the literature. The etiology of LCA remains unclear, and preliminary investigations indicate a potential association with immune system dysfunction and other visceral disorders (3). Given the rarity of reported LCA cases, especially isolated pediatric cases, robust evidence for elucidating its pathogenesis remains lacking, and further investigation requires the accumulation of more clinical cases and in-depth basic research (4). Clinically, most LCA patients are asymptomatic and diagnosed incidentally during physical examination or imaging for other conditions. A minority may present with nonspecific symptoms such as upper abdominal discomfort, abdominal pain anemia, thrombocytopenia, splenomegaly, or hypersplenism, there are also reports of concurrent malignant tumors (4, 5). In this case, the child only had slightly elevated platelets and splenomegaly without other specific symptoms, which is consistent with the clinical characteristics of asymptomatic LCA.

### 3.2 Imaging features and differential diagnosis

LCA has relatively characteristic CT findings, which are helpful for preoperative differential diagnosis (6). Isolated LCA typically presents as a large, round-like hypodense mass on CT plain scan, with uniform density, clear boundary, and local protrusion beyond the splenic contour. The characteristic enhanced CT manifestations include: (1) Arterial phase: Obvious heterogeneous enhancement resembling a “fireball” with density significantly higher than normal splenic parenchyma, which is related to the abundant blood supply of the tumor; (2) Portal venous phase: Continuous centripetal progressive enhancement, with density tending to be uniform; (3) Delayed phase: Uniform enhancement with mild attenuation, but still hyperdense to normal splenic parenchyma (6, 7). These imaging features are consistent with the findings of this case, which can help distinguish LCA from other splenic tumors. Although MRI was not performed in this case due to parental concerns, recent studies have confirmed that MRI is a valid complementary diagnostic modality for LCA. It exhibits high soft tissue resolution, with LCA typically showing hypointensity on T1WI and hyperintensity on T2WI, displaying uneveninternal signals mixed with speckled low signals, known as thespeckled and progressive centripetal enhancement similar to CT in the dynamic contrast-enhanced phase (6, 7). This characteristic MRI performance can further improve the accuracy of preoperative differential diagnosis.

The main differential diagnoses of isolated LCA include:

(1) Splenic cavernous hemangioma: The most common primary splenic benign tumor. On CT plain scan, it presents as hypodense nodules or masses, and some may have calcification (characteristic “phlebolith” sign). On enhanced scan, it shows peripheral nodular enhancement in the arterial phase, gradually filling in centripetally in the portal venous and delayed phases, which is different from the “fireball-like” enhancement of LCA. In addition, calcification is not reported in LCA, which can be used as a differential point.The tumor consists of endothelial cells and are positive for endothelial markers including CD31 and CD34 and negative for CD8, CD21, and CD68 (8); (2) Splenic hamartoma (SH): A rare, benign vascular proliferation. On plain and contrast-enhanced CT images, SH usually appeared as heterogeneous masses, sometimes containing areas of lower-density suggesting necrosis or hemorrhage and high-density calcifications.Postcontrast images usually showed diffuse homogenous or heterogeneous enhancement, indicating the vascular nature of the lesions.SHs consist of an abnormal proliferation of splenic red pulp, characterized by irregularly arranged vascular channels of varying caliber lined by splenic sinus endothelial (littoral) cells and surrounded by fibrous splenic (Billroth) cords, are positive for CD8, CD31, vimentin, and factor VIII (9); (3) Splenic angiosarcoma: pediatric cases are extremely rare, showing irregular tumor margin, obvious invasion of adjacent tissues on CT, and heterogeneous enhancement with rapid washout in delayed phase, accompanied by hemorrhage and necrosis. The tumor predominantly consist of sheets of spindled cells with nuclear atypia, are positive for CD31, ERG, factor VIII, and CD34 (10); (4) Splenic abscess: with typical clinical symptoms of fever and abdominal tenderness, showing ring enhancement with low-density necrotic center on CT, and the enhancement degree is significantly lower than LCA; (5) Pediatric splenic metastasis: almost secondary to advanced malignant tumors, with multiple lesions and mild enhancement in all phases, accompanied by abnormal tumor markers (11).

When the imaging findings of splenic space-occupying lesions in children are atypical (e.g., no typical “fireball-like” enhancement, unclear tumor margin, accompanied by abnormal laboratory indicators), imaging alone is insufficient for definitive diagnosis, and ultrasound-guided percutaneous core needle biopsy is recommended to obtain pathological evidence and avoid unnecessary surgical intervention.

### 3.3 Pathological diagnosis

It hard to diagnose LCA patients prior to surgery, and postoperative pathology is crucial to clarify it. Most of the gross specimens of isolated lesions show varying degrees of splenomegaly. The surface or sections of the spleen present nodular changes.The typical morphological features of LCA are anastomosing vascular lumens with varying degrees of dilation, which may be accompanied by papillary projections or cystic dilatation, and the tumor cells are flattened or cuboidal, with mild to moderate nuclear pleomorphism., tumor cells The most characteristic immunophenotypic feature of LCA is dual expression of endothelial markers (CD31, CD34) and histiocytic markers (CD68, CD163) (7), which is an important basis for distinguishing LCA from other splenic vascular tumors. In this case, immunohistochemical staining revealed positive expression of CD31, CD34, CD68, and CD163 in tumor cells, consistent with the typical immunophenotype of littoral cell angioma (LCA) and confirming the diagnosis. In contrast, tumor cells were negative for both CD20 and CD3, distinguishing them from normal splenic tissue that harbors abundant CD20- and CD3-positive lymphocytes.

### 3.4 Treatment and prognosis

The main treatment for LCA is surgical resection, including total splenectomy and partial splenectomy (12). Total splenectomy is traditionally recommended for multiple lesions or large tumors involving the entire spleen, while partial splenectomy is preferred for isolated lesions to preserve partial splenic function, especially in pediatric patients, as splenectomy may increase the risk of postoperative sepsis, thrombosis, and other long-term complications (13). In this case, laparoscopic partial splenectomy was performed, and the child recovered well without complications or recurrence during follow-up, which is consistent with the favorable prognosis of isolated LCA.

The prognosis of LCA is generally good. A recent analysis of 435 patients showed that LCA is a benign tumor with stable disease or slow growth, and the recurrence rate after surgical resection is low (2). However, some studies suggest that LCA may be associated with underlying malignancies, and long-term follow-up is needed to monitor for the occurrence of other tumors (5). For pediatric patients with isolated LCA, the prognosis is better due to the absence of underlying diseases and better tissue repair ability, but regular postoperative follow-up is still required. Due to the rarity of pediatric LCA, there is currently no specific literature or guidelines to guide follow-up schedules. Therefore, the follow-up strategy is based on our institutional clinical management experience in treating pediatric splenic benign tumors, with a focus on balancing thorough surveillance and avoiding over-medicalization. The follow-up protocol is not a generalized recommendation but rather an individualized approach derived from our institutional clinical experience:(1)Short-term follow-up (0~6 months after surgery) was performed monthly for the first 3 months and then every 3vmonths for the subsequent 3 months. Follow-up assessments included clinical physical examination to evaluate abdominal symptoms and signs of infection, as well as abdominal ultrasound to screen for local recurrence, thereby avoiding radiation exposure. This schedule was designed to monitor early postoperative recovery and exclude acute complications; (2)Medium-term follow-up (7~12 months after surgery) was conducted every 6 months. Surveillance consisted of clinical evaluation and abdominal ultrasound as the primary imaging modality. CT or MRI was not performed unless abnormal findings were detected on ultrasound or clinical examination, to avoid unnecessary radiation exposure and ensure continuous monitoring for tumor recurrence; (3)Long-term follow-up (more than 1 year after surgery) was carried out every 6~12 months. Assessments included clinical physical examination and abdominal ultrasound. If suspicious symptoms occurred, such as abdominal pain or unexplained fever, low-dose CT was performed for further clarification while minimizing radiation exposure.

## 4 Limitations

This study is a single-center case report with a small sample size, and the conclusions need to be verified by multi-center, large-sample studies. In addition, the long-term prognosis of pediatric LCA remains unclear, and longer follow-up is needed to evaluate the recurrence rate and long-term complications. A limitation of this study is that high-power magnification histological images could not be provided, and the image quality represents the highest achievable level from our institutional archived pathological data, which may affect the readers’ detailed observation of the tumor’s histological features. The absence of MRI examination is a also major limitation of this study. Although CT has shown typical enhancement features, MRI with high soft tissue resolution can provide more detailed information on tumor parenchymal signal and vascular distribution, which is helpful for further differential diagnosis with splenic hemangioendothelioma. The future studies should combine multi-modality imaging to improve the diagnostic accuracy of pediatric LCA.

## 5 Conclusions

(1) Isolated splenic littoral cell angioma in pediatric patients is extremely rare, with nonspecific clinical manifestations. CT imaging has characteristic features (“fireball-like” arterial enhancement, progressive uniform enhancement in portal venous and delayed phases), which are valuable for preoperative indication. Pathological examination with dual immunophenotypic expression (CD31+, CD34+, CD68+, CD163+) is the gold standard for diagnosis. (1) It is the first reported case of isolated pediatric LCA (≤9 years old) with complete dynamic CT enhancement data and pathological correlation of the “fireball-like” enhancement pattern; (2) The application of laparoscopic partial splenectomy with a 1.5 cm tumor margin in this young child enriches the surgical experience of minimally invasive treatment for pediatric splenic benign tumors; (3) The individualized tiered follow-up strategy combined with pediatric radiation protection requirements fills the gap of standardized follow-up plans for pediatric LCA in current literature.Accumulating clinical, imaging, and pathological experience is crucial for improving the diagnosis and treatment of this rare disease.

## Data Availability

The original contributions presented in the study are included in the article/supplementary material. Further inquiries can be directed to the corresponding author.
